# Background and Recent Advances in the Locata Terrestrial Positioning and Timing Technology

**DOI:** 10.3390/s19081821

**Published:** 2019-04-16

**Authors:** Chris Rizos, Ling Yang

**Affiliations:** 1 School of Civil and Environmental Engineering, University of New South Wales, Sydney 2052, Australia; c.rizos@unsw.edu.au; 2 College of Surveying and Geo-Informatics, Tongji University, Shanghai 200092, China

**Keywords:** Locata, PNT, terrestrial constellation

## Abstract

Global Navigation Satellite System (GNSS) is the most widely used Positioning, Navigation, and Timing (PNT) technology in the world today, but it suffers some major constraints. Locata is a terrestrial PNT technology that can be considered as a type of localised “constellation”, which is able to provide high-accuracy PNT coverage where GNSS cannot be used. This paper presents a comprehensive literature review of the Locata technology and its applications. It seeks to answer questions, such as: (1) What is Locata and how does it work? (2) What makes Locata unique compared with other terrestrial positioning systems? (3) How has Locata been used in different applications for accurate PNT? (4) What are the current challenging issues that may restrict its further adoption for custom-grade navigation in urban environments?

## 1. Introduction

The field of Positioning, Navigation, and Timing (PNT) has been an active research and development focus for many decades. The Global Navigation Satellite System (GNSS) is undoubtedly the most widely used PNT technology today, with user equipment and techniques having evolved over the past few decades to their current highly-developed forms. Despite this success, GNSS cannot satisfy the PNT requirements in many commonly traversed environments, such as in urban areas and indoors. A variety of technologies have been developed to compensate for such limitations, which include short-range Radio Frequency Identification (RFID), Ultra-Wideband (UWB), Zigbee, WiFi, Bluetooth, ultrasonic, new ranging systems that are based on pseudolites, image-based technologies, inertial sensors for dead-reckoning, magnetic-based, proximity sensors, mobile telephony, etc. However, there is no technology that can be used independently, or together with GNSS and other systems, which has the advantages of GNSS, and in particular can deliver accurate PNT solutions that are similar to advanced and augmented GNSS techniques, such as Differential GNSS (DGNSS) or Precise Point Positioning (PPP).

A number of researchers have claimed that pseudolites can be used to enhance PNT availability, reliability, integrity, and accuracy in applications, such as aircraft approach and landing [1,2], deformation monitoring applications [3], Mars exploration [4], and others [5,6,7,8,9]. However, extensive research and testing has concluded that pseudolites have fundamental technical problems that, even in a controlled or lab environment, are extremely difficult to overcome [7,8,10,11]. The challenges of optimally siting pseudolites, controlling transmission power levels, overcoming “near-far” problems, trying to ensure extremely high levels of time synchronisation, configuring special antennas, and designing the “field of operations”, such that GNSS and pseudolites can work together (or at least not interfere with each other), have been largely insurmountable in the real world [12].

Other solutions have been sought, due to these difficulties. One is the “Locata” concept that is based on pseudolite principles but with significant enhancements. Locata Corporation developed the Locata technology, and it has evolved since 1995 with academic collaboration of the University of New South Wales (UNSW Sydney) and the University of Nottingham (UK). Locata is a terrestrial Radio Frequency (RF)-based system that has several unique characteristics which make it superior to conventional pseudolite-based systems [13,14,15]. This paper presents a literature review of the Locata technology and its applications, while also discussing its main challenges. The rest of the paper is structured, as follows. Section 2 describes the core elements of the Locata technology. Section 3 presents the development history and mathematical models for Locata precise positioning. In Section 4, some PNT applications for which the Locata technology has been implemented are reviewed. Section 5 discusses the main challenges that may restrict its future application for navigation in urban environments. Finally, Section 6 presents concluding remarks and recommendations.

## 2. Locata Core Technologies

The Locata concept has been designed to overcome the limitations of GNSS, as well as other pseudolite-based positioning systems, with four key objectives: (1) Being available in all environments; (2) High reliability; (3) High accuracy; and, (4) Cost effectiveness [16].

**Figure 1:**
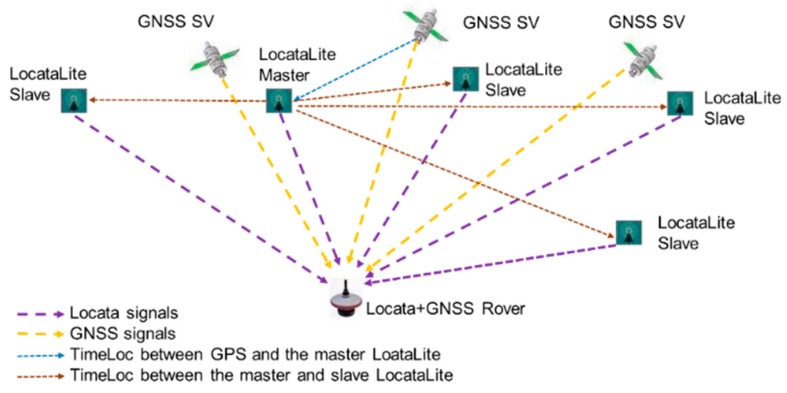
The Locata technology positioning concept.

In the Locata concept, these objectives are achieved through a design based on a network (a “LocataNet”) of ground-based *transceivers* (“LocataLites”) that cover a chosen area with strong signals, which is suitable for accurate positioning in classically difficult GNSS environments (Figure 1). Importantly, the positioning signals that are emitted by different LocataLites are time-synchronised (via “TimeLoc”) to the order of 30 picoseconds, which allows a single mobile Locata receiver (“Locata Rover”) to determine its position within the network in the same manner as GNSS. However, unlike GNSS, the sub-centimetre level of synchronisation between LocataLites allows for single-point positioning with GNSS Real-time Kinematic (RTK) level of accuracy, *without the use of a reference station, in a manner unlike GNSS Precise Point Positioning*. The Locata Rover can operate in an environment where GNSS signals augment the Locata system (and visa versa), or they can operate entirely independently of GNSS [16]. 

### 2.1. LocataNet

A LocataNet is a terrestrial network of synchronised LocataLites that provides positioning signals that support a range of PNT applications. Barnes et al. summarise the characteristics of the LocataNet [17,18]. When building a LocataNet the location of the LocataLites should satisfy the requirements of: (1) be able to receive the signal from at least one other LocataLite; and, (2) the geometry of the network (as indicated by a measure such as the Dilution of Precision—DOP) is suitable for PNT application. 

In general, a LocataNet can be established in any environment, and could be optimised for power source, power transmission, DOP, security of the LocataLites, and other factors. In a Locata applied environment, the elevation angles are usually low, which typically makes vertical DOP (VDOP) significantly larger than the horizontal DOP (HDOP) values [19,20,21]. LocataLites must be distributed in such a way that there are sufficiently large height differences between pairs of LocataLites to achieve better VDOP. In general, it is considered that the LocataNet geometry (location and number of LocataLites) can be optimised beforehand to satisfy the positioning accuracy and reliability that are required by specific applications. However, optimisation is only guaranteed without any misspecification in the function and as long as the assumptions underlying the mathematical model hold, especially the stochastic model. Previous studies typically assumed a unit weight for all code pseudorange or carrier phase measurements, regardless of the spatial and temporal heterogeneity of the signals [22,23]. Therefore, the prior quality control of a LocataNet is still problematic, unless the Locata signals’ stochastic characteristics are theoretically investigated and accurately specified.

### 2.2. TimeLoc

Locata enables precise positioning by its high accuracy timing capability, which is referred to as TimeLoc technology. TimeLoc is a wireless synchronisation methodology that achieves time synchronisation to better than about 30 picoseconds among multiple LocataLites, without the need for expensive atomic clocks, external cables, or a master reference receiver [16]. Barnes et al. [17,18,24] and Crawford [25] provide a brief description of the TimeLoc procedure. Importantly, during the synchronisation procedure, the geometrical offset between two LocataLites must be precisely corrected for, which requires: (1) careful installation of LocataLites to avoid multipath; (2) precise survey of the coordinates of the antennas of each LocataLite; and, (3) accurate correction for the tropospheric delays. As shown in Figure 1, the master LocataLite can also be locked on to an external GNSS time, so that the LocataNet is seamlessly integrated with a GNSS constellation.

### 2.3. LocataLite

**Figure 2:**
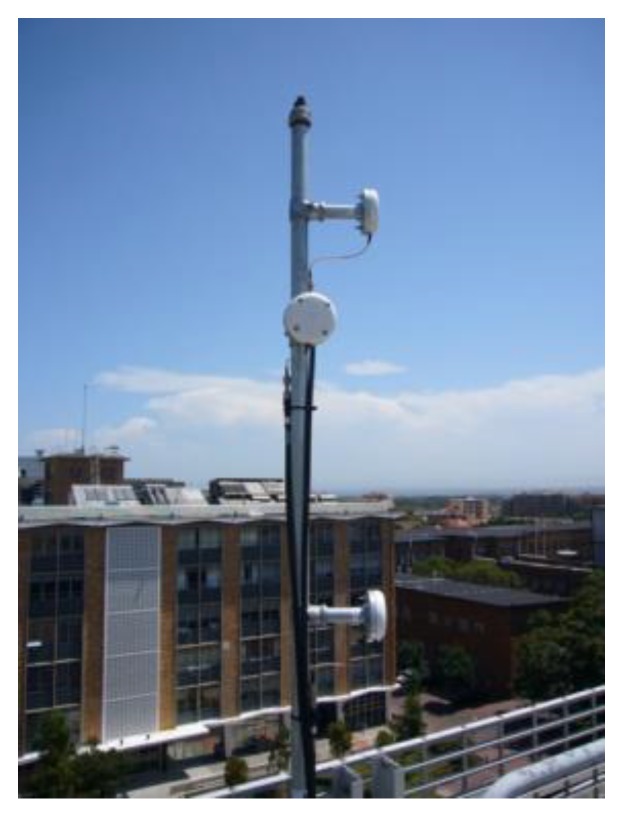
Typical LocataLite setup [29].

A LocataLite is a transceiver device that is deployed in a Locata network that replicates a GNSS constellation’s functions—but locally and on the ground. It currently transmits Code Division Multiple Access (CDMA) signals on two frequencies within the 2.4 GHz Industrial Scientific Medical (ISM) band (approximately 2.41428GHz and 2.46543GHz, referred to as S1 and S6 respectively), and from two spatially-separated antennas, which produce four ranging signals. The use of signals in the 2.4 GHz ISM band allows for (nearly) worldwide deployment without the need for RF signal transmission licences, and it does not impinge on the GNSS L-band signals [26]. For centimetre-level accuracy positioning, these signals must exceed the Locata Signal Strength (LSS) threshold of 5 [16,27]. To address the multipath signal fading effects, the Locata hardware designs employ three types of signal diversity implementation: spatial, polarisation and frequency diversity [28]. Figure 2 shows a typical antenna configuration for a LocataLite, with one receiving (middle) and two transmitter antennas, which are separated vertically. The transmitter antennas are angled to point into the coverage area, and the receive antenna pointing at the master LocataLite to ensure time synchronisation [20].

The Locata signals are closely related to those of the Global Positioning System (GPS), making integration with a GPS receiver easier, at least in theory. The receiver and the transmitter chipset share the same clock, which is a cheap temperature-compensated crystal oscillator (TCXO), by which the Locata signals are digitally generated and can be operated in a Pulse Amplitude Modulation (PAM) with different duty cycles, power output, and Pseudo-Random Noise (PRN) type codes. PAM is commonly used with pseudolite signals (instead of a continuous transmission, as for GNSS) to reduce interference and increase the working range (to overcome the “near-far” problem). “Duty cycle” refers to the percentage of time spent transmitting a signal when in PAM. In principle, LocataLites can be designed to broadcast at a range of microwave frequencies, modulated with digital codes of any design, at any power deemed to be appropriate for a specific application.

### 2.4. Locata Rover

A Locata Rover is a mobile or user receiver whose output is a PNT solution normally expected of a GNSS receiver. Currently, the Locata Rover (like the LocataLite) uses Field Programmable Gate Array (FPGA) technology in a modular hardware design with separate receiver and RF transmission boards. It is approximately half the size of the LocataLite, since the transmitter board is not required. Raw measurement data (code pseudorange and carrier phase) from the receiver can be obtained. Real-time positioning rates of up to 25Hz are possible by streaming the receiver data to the Locata Integrated Navigation Engine (LINE) application, which runs on either an external computer or an internal processor.

The Locata Rover and LINE use a Direct Carrier Ranging (DCR) algorithm to determine its position and timing from measurements that were made to at least four (for three-dimensional (3D) positioning) or three (for two-dimensional (2D) positioning) LocataLites. This algorithm is similar to that of standard GNSS Single Point Positioning (SPP), but it uses carrier phase measurements instead [17]. In order to perform DCR, the carrier phase ambiguities must first be resolved. Currently, this is done either via the Known Point Initialisation (KPI) method or an On-the-Fly (OTF) procedure [16].

### 2.5. VRay Antenna

**Figure 3:**
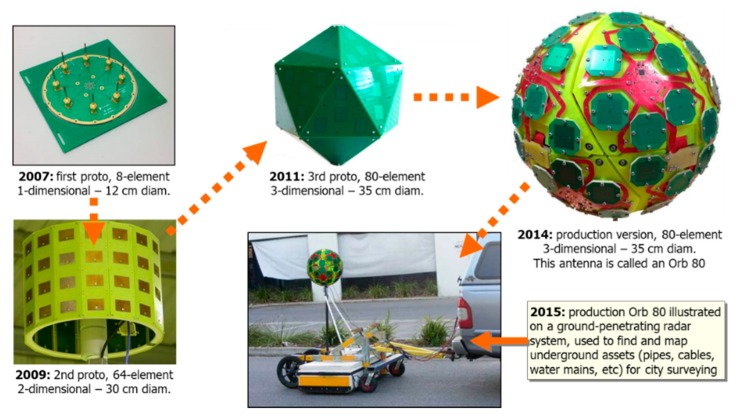
The prototypes and current production model of the Locata beam-forming VRay antenna.

VRay is an antenna that is designed for indoor and outdoor industrial applications, which mitigates multipath using a Correlator Beam-Forming (CBF) technique [26]. To mitigate multipath, the beams that formed at the receiver must be pointed to the directions of the LocataLite transmitters. The receiver initially sweeps the beams in a search pattern so as to maximise the signal power from each LocataLite. Once the optimal beam settings are determined, the corresponding angle-of-arrival measurements are used to estimate both the approximate position of the Locata Rover receiver and the antenna orientation [30]. Figure 3 shows the evolution in the development of the VRay antenna.

As shown in Figure 3, the first VRay prototype was developed in 2007, and it was a one-dimensional 12 cm diameter board with eight elements. The second prototype developed (2009) was a two-dimensional 30 cm diameter device with 64 elements. The last prototype (2011) had a three-dimensional form with 80 elements. The commercial version (2014) is a basketball-sized spherical array that consists of 20 panels with 80 elements. Each panel comprises four elements arranged with one central element and the three other elements forming a triangle. The element spacing between each panel is half the signal wavelength [26]. The results from both static and kinematic experiments have confirmed that the positioning accuracies are at the centimeter-level, and the antenna orientation solutions are accurate to a few degrees [30,31,32].

## 3. Locata Development History and Mathematical Models

### 3.1. Locata’s Prototype and Second Generation System

**Figure 4:**
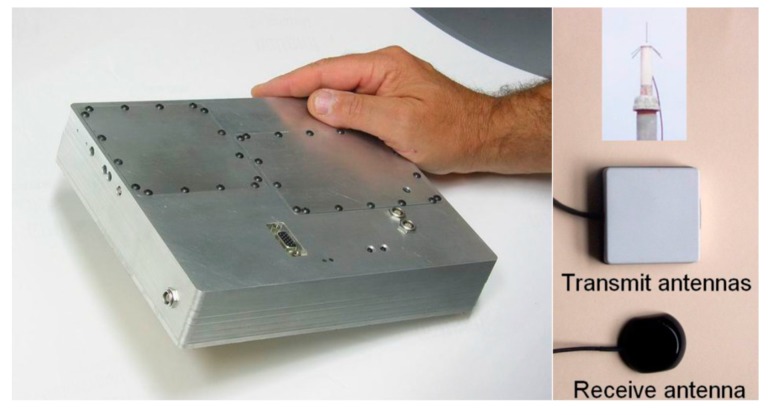
First generation LocataLite hardware and antennas [18].

A prototype system had been built and tested between 2002 to 2005 as a proof-of-concept demonstration of the Locata positioning technology, and to verify key system concepts and algorithms. This first generation system used single-frequency transceivers (LocataLites) and a modified standard GPS receiver as the mobile unit (the Locata Rover receiver). Commercially available GPS patch antennas were used for the receiver and transmitter, in addition to a custom built ¼ wave antenna for one of the LocataLite transmitters, as shown in Figure 4.

Although the proof-of-concept was successfully demonstrated, the Locata system had some significant limitations, including: (1) the challenge of interoperability with GPS—due to the prototype Locata signal transmissions being at the GPS L1 frequency; (2) OTF carrier phase ambiguity resolution based on KPI; (3) limited multipath mitigation—using a standard GPS L1 C/A code signal structure; and, (4) limited transmitter range and penetration—limited transmission power allowed in the GPS L1 frequency band, so as to not interfere with GPS signals [16,18].

To address these limitations Locata designed and built a second generation system, which is the current commercially available system, as described in Section 2. This new design incorporates Locata’s own proprietary signal transmission structure, different transmission frequencies, and allows the complete control over both the signal transmitter and receiver, offering enormous flexibility. Table 1 provides a summary of Locata’s prototype and second generation system.

### 3.2. Locata Mathematical Model

#### 3.2.1. Locata Measurements

Similar to GNSS, the range measurements of Locata are of two types: code pseudorange and carrier phase. The carrier phase measurements are more precise than the code pseudorange measurements. The basic Locata observation equation between receiver and LocataLite channel *i* can be written as:

(1) $$\{\begin{array}{c} P_{j}^{i}=\rho^{i}+\tau_{trop}^{i}+c\cdot \delta t+\varepsilon_{P,j}^{i}\\ \Phi_{j}^{i}=\rho^{i}+\tau_{trop}^{i}+c\cdot \delta t+\lambda_{j}\cdot N_{j}^{i}+\varepsilon_{\varphi ,j}^{i} \end{array}$$

where $P_{j}^{i}$ and $\Phi_{j}^{i}$ are the code pseudorange and carrier phase measurements, respectively (in units of metres), $\rho^{i}$ is the geometric distance between the Locata Rover and the transmitting antenna *i*, $\tau_{trop}^{i}$ is the tropospheric delay, *c* is the speed of microwave signals in a vacuum, δt is the receiver clock bias, $\lambda_{j}$ is the wavelength of the *j*^th^ frequency, $N_{j}^{i}$ is the carrier phase ambiguity term, and $\varepsilon_{P,j}^{i}$ and $\varepsilon_{\varphi ,j}^{i}$ are the lumped sum of unmodelled residual errors (including noise) on code pseudorange and carrier phase measurements, respectively. Note that there is no transmitter clock bias in the observation equation because of the tight time synchronisation of the LocataLites. Nor is there an ionospheric delay term. Unlike GNSS, Locata’s ambiguities are typically estimated as the floating-point values [33].

The receiver clock bias can be estimated or eliminated using Single-Differenced (SD) measurements between the two LocataLite channels. Similar to GNSS SD between satellites, the SD measurements for the Locata system are:

(2) $$\{\begin{array}{c} \Delta P_{j}^{ik}=P_{j}^{i}-P_{j}^{k}=\Delta \rho^{ik}+\Delta \tau_{trop}^{ik}+\Delta \varepsilon_{P,j}^{ik}\\ \Delta \Phi_{j}^{ik}=\Phi_{j}^{i}-\Phi_{j}^{k}=\Delta \rho^{ik}+\tau_{trop}^{ik}+\lambda_{j}\cdot \Delta N_{j}^{ik}+\Delta \varepsilon_{\varphi ,j}^{ik} \end{array}$$

where “Δ” denoting the single-differencing operation between two LocataLite measurements at the receiver. For precise positioning, the SD tropospheric delay should be corrected for using an appropriate tropospheric model, or estimated as an additional state in the Kalman filter [21,34]. 

#### 3.2.2. Precise Positioning Methods

The Locata SD carrier phase measurements are used for precise positioning, with the float ambiguities being resolved either by the KPI or OTF techniques. KPI is implemented for LocataLite channel *i* by the following set of equations

(3) $$\lambda_{j}\cdot \Delta N_{j}^{ik}=\Delta \Phi_{j}^{ik}-\Delta \rho^{ik}-\Delta \tau_{trop}^{ik}-\Delta \varepsilon_{\varphi ,j}^{ik}$$

The initial position of the Locata Rover is required to calculate the precise $\Delta \rho^{ik}$. A surveying instrument, such as a “total station” or a geodetic-grade GNSS, can be used to compute accurate initial coordinates [35]. Once the floating ambiguities are estimated with a certain level of accuracy, they can be treated as ‘fixed’ known parameters in Equation (2).

OTF ambiguity resolution is dependent on transmitter–receiver geometry *change*. Although the LocataLites are fixed on the ground, the trajectory of the Locata Rover provides the spatial diversity in the measurements, which makes the OTF ambiguity resolution possible. OTF ambiguity resolution can be implemented either via an Extended Kalman Filter (EKF) [23,29,36] or a nonlinear batch Least Squares (LS) algorithm [37] using SD carrier phase measurements. However, for applications, such as continuous displacement monitoring, the Locata Rover is static, and the KPI method is more useful.

#### 3.2.3. Locata Integrated with GNSS/INS

During the development of Locata’s prototype system, the main objective was to develop the proof-of-concept software and hardware for the LocataLite, the LocataNet, and the Locata Rover. In many applications, there may be less than four LocataLites in a LocataNet. Therefore, integrating GNSS and Locata measurements to provide a more accurate and reliable position and navigation solution in GNSS-deficient environments has been investigated for applications, such as open-cut mining, structural deformation monitoring, and aircraft landing [1,10,11,17,38,39,40,41,42].

More recently, researchers have investigated “triple integration” of Locata, GNSS, and Inertial Navigation System (INS) measurements for PNT solutions. Locata is straightforward in integrating Locata with GNSS, or to extend the traditional GNSS/INS integration system to a Locata/GNSS/INS triple-integration system since it relies on GNSS-like range measurements to generate position and navigation solutions. For a Locata/GNSS/INS system, the INS error equations define the dynamic model [43], whereas the measurement model contains measurement information from the GNSS and Locata systems. Classifying according to measurement models used, there are loosely-coupled, cascaded-coupled, and tightly-coupled integration architectures [44]. Classifying by filter structures, the Centralised KF (CKF), Decentralised KF (DKF), Parallel KF (PKF), Federated KF (FKF), and Global Optimal Filtering (GOF), have been investigated for marine, aviation, and land vehicle navigation applications [45,46,47].

#### 3.2.4. Measurement Errors and Biases

(1) Clock biases

There is no transmitter clock bias present in the measurement due to the tight time synchronisation of the LocataLites. However, the receiver clock is not synchronised to the LocataNet and therefore is subject to drift. The receiver clock offset has either to be introduced as an additional unknown into the LS estimation algorithm or eliminated by forming SD observables [33,35,37].

(2) Cycle slips

Cycle slips may occur in the Locata carrier phase measurement due to signal loss, signal obstructions, or internal receiver tracking problems. It has been reported that Locata cycle slips may be multiples of half cycles [35].

(3) Tropospheric delay

Traditional GNSS tropospheric models cannot be used since the Locata signal travels very close to the Earth’s surface [48]. Microwave-based Electronic Distance Measurement (EDM) tropospheric correction models are recommended, such as the RTCA (2000) model, the Werner model [49,50], the Bouska and Raquet model [34], the local differential corrections model [51], as well as the length-based correction models for EDM [52,53]. 

(4) Multipath

Multipath is a serious error source due to the low elevation angles in a typical Locata scenario [21]. Bonenberg et al. investigated the influence of issues, such as different power levels and shielding options (metal plate and a choke ring) [54]. Nowadays, using the VRay antenna is perhaps the best option for mitigating against multipath disturbance.

## 4. Locata Applications

Locata technology provides centimetre-level accuracy positioning that could complement, or even replace, conventional GNSS in classically difficult GNSS signal environments, such as open-cut mines, deep valleys, heavily forested areas, urban environments, and even indoor locations [55].

### 4.1. Open-Cut Mining

Barnes et al. [56] described an integrated Leica/Locata system for open-cut mining operations. In this loosely-coupled system, the Leica GNSS-RTK receiver was used to initialise the Locata receiver’s position. Following this initialisation, Locata operated independently of GNSS, and it demonstrated accuracies that were comparable to GNSS-RTK.

From late 2006 through to early 2007, the first commercially available integrated Leica and Locata devices were installed and tested at DeBeers Venetia Mine in South Africa. Leica/Locata enabled positioning technology was installed on drill, dozer, and backpack systems. Extensive data analysis of data collected over two months showed that the overall positioning accuracy was within 10 centimetres in a horizontal sense, and 20 centimetres vertically [40].

### 4.2. Deformation Monitoring

Later, the performance of the 2nd generation Locata technology for structural deformation monitoring was assessed using a LocataNet that was established at the UNSW Sydney campus for a long-term static test and a simulated deformation movement test [41]. In [35], a series of deformation trials using the Locata technology were carried out at the Tumut Pond Dam (Cabramurra, Australia). These analyses confirmed that the Locata technology could generate positioning results with millimetre-level accuracy. Bonenberg et al. [57] further demonstrated Locata’s performance in a five-day monitoring trial, comparing it against GNSS-RTK.

### 4.3. Flight Tests

Augmenting GPS and/or INS for flight navigation is a primary application of Locata technology. Technical issues, such as antenna offsets between different systems, the influence of the number, location and geometric distribution of the LocataLites on ambiguity resolution, positioning accuracy, system reliability, and stability had been analysed via a series of flight tests. At first by simulations [1], and subsequently by real world tests that were carried out at Wedderburn Airfield, Australia [58], and south of Canberra, Australia [59]. A series of Unmanned Aerial Vehicle (UAV) flight tests were also conducted at the Advanced Navigation Technology Center, at the US Air Force Institute of Technology [29]. These analyses confirmed that Locata real-time positioning accuracy was at the centimeter-level, and that augmenting Locata with GPS can significantly improve the vertical positioning accuracy. 

In October 2011 another flight trial was conducted, from Bankstown Airport to Cooma Snowy Mountains Airport, in Australia. Utilising data from airborne sensors, including dual-frequency GPS receivers, GPS/INS integrated systems, and Locata Rover receivers, the investigations were conducted on the positioning performance of a Locata-alone system, Locata/GPS integration systems, and triple-integrated Locata/GPS/INS systems [37,47,60].

### 4.4. Marine Navigation

Sydney Harbour by Locata Corporation, UNSW Sydney, and several New South Wales government agencies conducted a field trial in October 2012 to demonstrate Locata’s positioning capability in harbour areas. Experiment analyses that were focused on Locata carrier phase processing and Locata/INS loosely-coupled integration systems [33], on GPS/Locata precise point positing [23], and on different data fusion algorithms applied to triple-integrated GPS/Locata/INS systems [45]. However, the publications that focused on evaluating the positioning performance at the central harbour areas where the Locata signals were relatively stable and there was high availability. Section 5.2 presents a detailed analysis of the Locata signal spatial characteristics and the positioning performance diversity within the whole test area. 

### 4.5. Indoor/Outdoor Vehicle Tracking

Locata’s application on vehicle tracking and precise positioning were conducted both indoors and outdoors. In the beginning, static/kinematic positioning was implemented through an integration of GPS and Locata, due to the lack of redundancy of Locata measurements. Tests of using prototype Locata technology demonstrated that outdoor static and kinematic positioning accuracy were at the sub-centimetre-level and centimetre-level, respectively. Due to errors that were induced by possible multipath and obstructions, indoor kinematic positioning accuracy was reduced to the sub-metre-level [10,17,39,61]. Later in [62], the feasibility of using Locata for industrial machine guidance was demonstrated through a trial at BlueScope Steelworks under an extremely severe multipath environment, where the positioning results were with sub-centimetre precision and centimetre-level absolute accuracy. More recently, fault-tolerant decentralised GNSS/Locata/INS integration algorithms were investigated for land vehicle navigation [46]. To mitigate the multipath effects, VRay antenna was designed and applied for vehicle tracking, and investigations regarding the positioning algorithms were detailed in [31,32].

### 4.6. Locata Working Independently, or in Combination with GNSS/INS

As can be seen from the descriptions above, all of these applications treat Locata as a type of localised RF signal “constellation”, which is able to provide signal coverage to support high accuracy positioning where GNSS may not be available. Locata can also be tightly-coupled with GNSS due to its accurate external time synchronisation capability. This enhances the geometric strength and measurement redundancy. In early applications using Locata’s prototype system, investigations focused on either augmenting the GPS solution with additional Locata measurements or verifying the precise positioning capability of the Locata standalone system. More recently, academic research has focused on integrating Locata with GNSS and INS while using advanced data fusion algorithms and architectures. Multi-sensor integration is necessary for a variety of seamless indoor/outdoor positioning and navigation applications. The Locata technology has several advantages in high accuracy positioning: (1) TimeLoc allows the LocataNet to precisely synchronised (e.g. with respect to a GNSS time scale), so that the integration system can maintain a common time reference; (2) the VRay antenna provides angle measurements, which are rarely available from other navigation sensors; and, (3) the VRay antenna is highly effective in mitigating multipath signal disturbances.

## 5. Challenging Issues for Locata

### 5.1. RF Interference

A Locata network faces challenges in the presence of RF interference (RFI), since it operates in the licence-free 2.4 GHz ISM band. One potential interferer is WiFi signals, due to their use of the whole ISM band [63,64,65]. It had been confirmed that WiFi signals transmitted at different data rates have different effects on Locata performance. However, a remarkable improvement in RFI rejection was observed in Locata Version 3 devices by using dual-frequency carrier signals, dual-antenna transmissions, Time Division Multiple Access (TDMA) signal coding, and multiple carrier tracking loops [63]. The RFI can also be reduced by restricting the transmission time slots of the LocataLites [64], or by an adaptive inter-loop aiding scheme that helps to maintain lock in weak signal and moderate receiver dynamics environments [66]. However, research has also shown that high levels of narrow-band interference would still compromise the positioning performance [65]. In order to further reduce RFI, WiFi interference detection, as well as adaptively reweighting the Locata measurements, have been investigated, and shown to improve performance [67,68].

### 5.2. Raw Data Analysis of a LocataNet

**Figure 5:**
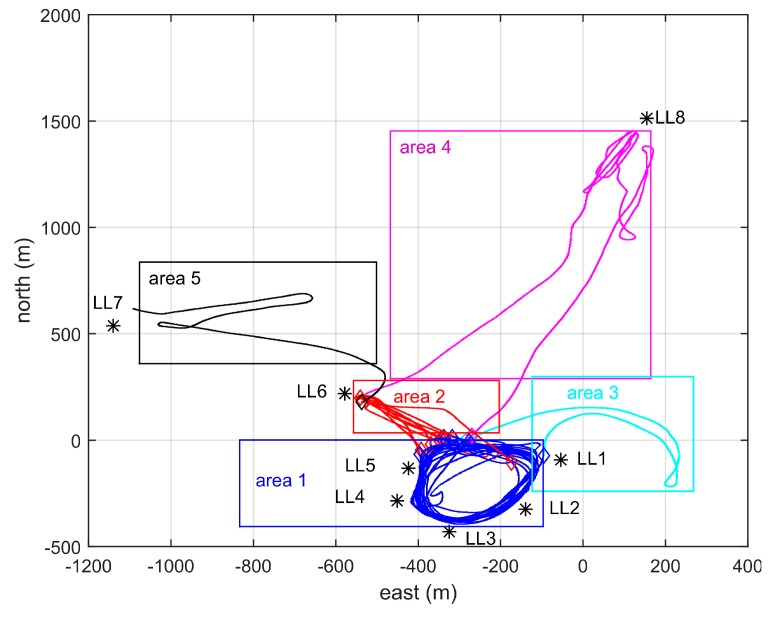
Location of LocataLites and experiment trajectory.

Locata technology can deliver metre-level positioning accuracy using code pseudorange measurements, and centimetre-level accuracy using carrier phase measurements. For the test THAT was conducted on Sydney Harbour in 2012, eight LocataLites (LL) were deployed in the LocataNet, with six installed along the shore (LL1–LL6), one on the Harbour Bridge (LL7), and another at Kirribilli (LL8). Figure 5 shows the location of the LocataLites and the Rover trajectory during the three hours experiment. To investigate the Locata signal quality, the trajectory was divided into five sections or areas, which are denoted by different colours in Figure 5. The Locata Rover trajectory was calculated from the GPS-RTK solution, and is thus accurate to the centimetre-level.

**Figure 6:**
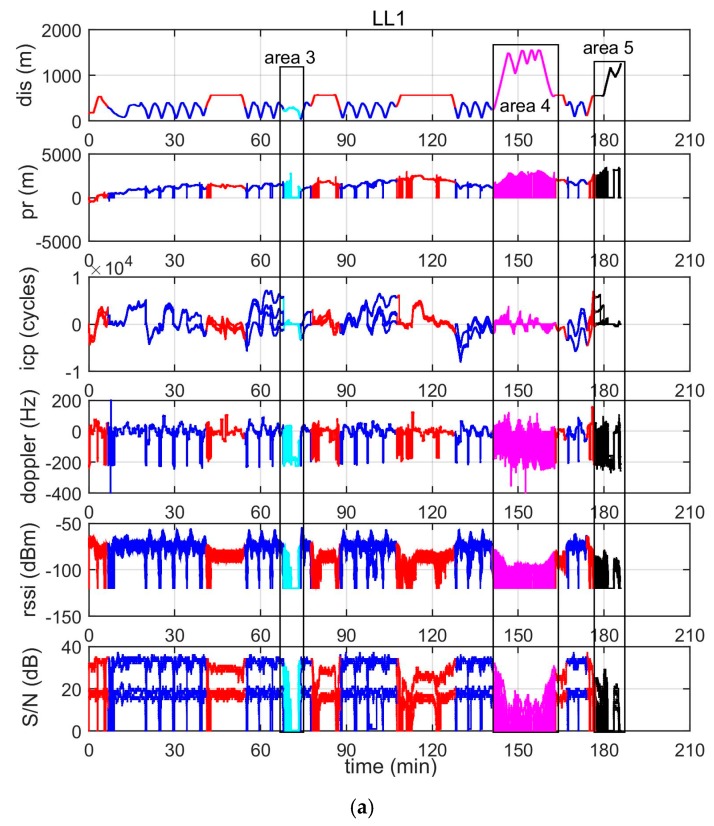

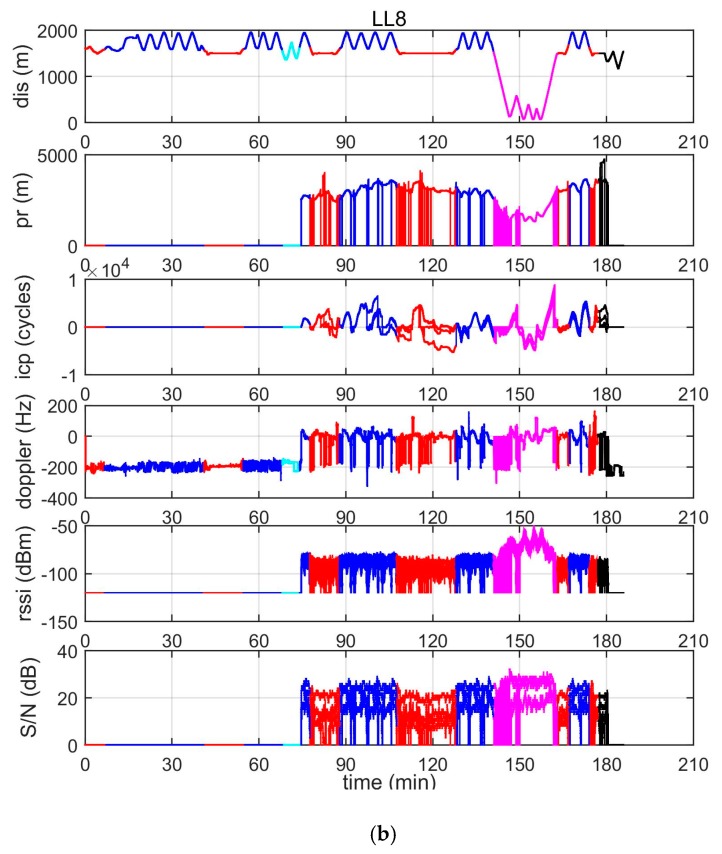
Raw measurements from LL1 (**a**) and LL8 (**b**).

Five types of measurements are made on a Locata Signal: code pseudorange in metres (pr), carrier phase in cycles (icp), Doppler frequency in Hz, received signal strength indicator in dBm (rssi), and signal-to-noise ratio (S/N) in dB. Figure 6 is a plot of the five types of raw measurements from LL1 (a) and LL8 (b), as well as the corresponding distance from the Rover to the LL (top panel). In each panel, four values from two antennas (Tx1, Tx2) at two frequencies (S1, S6) are plotted. Both of the subfigures show that there were frequent signal blockages or interruptions, both for LL1 and LL8 during the test, and signals within the areas 3, 4, and 5 are, in general, unavailable. Furthermore, the subfigure (b) shows that signals from LL8 are unavailable during the first 75 minutes, when the vessel travelled in areas 1 and 2.

**Figure 7:**
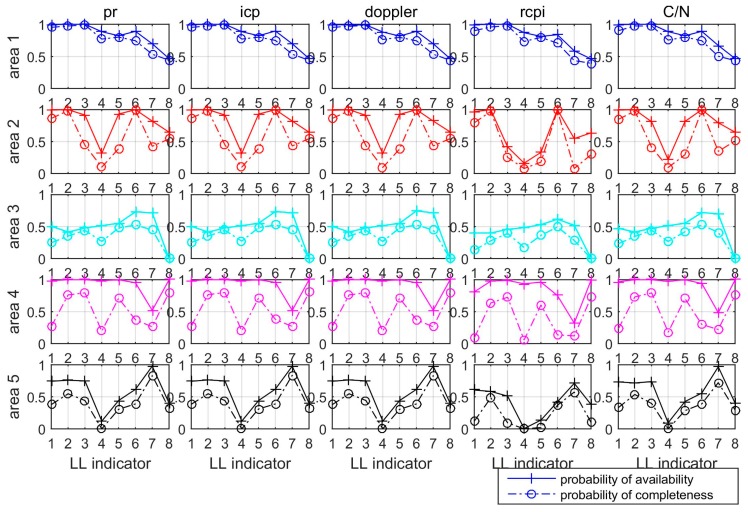
Probability of available measurements for each area for each LocataLites (LL).

To further analyse the signal availability for each LL, the probability of available measurements within each area for each LL are shown in Figure 7, where the solid line denotes the probability of availability, and the dash line denotes the probability of completeness, as defined by Equation (4). The probability of availability accounts for epochs when at least one measurement from the two antennas at the two frequencies is available, and the probability of completeness only counts epochs when the four measurements are all available. Figure 7 generally shows that using dual-frequency signals and dual antennas significantly improves the signal availability, especially within areas 2, 3, and 4, where the probability of availability is obviously higher than the probability of completeness for most LLs. Within area 1, the probabilities of availability is very similar to the probability of completeness for LL1, 2, 3, 5, and 8, and are nearly 10% higher for LL4, 6, and 7. In general, the LocataNet configuration provides optimal navigation performance within area 1 other than within area 2 to area 5.

(4) $$\left\{ \begin{array}{l} \mathrm{probability}\;\mathrm{of}\;\mathrm{availability}=\frac{\mathrm{epochs}\;\mathrm{of}\;\mathrm{at}\;\mathrm{least}\;1\;\mathrm{measurement}\;\mathrm{valid}}{\mathrm{total}\;\mathrm{number}\;\mathrm{of}\;\mathrm{epochs}}\\ \mathrm{probability}\;\mathrm{of}\;\mathrm{completeness}=\frac{\mathrm{epochs}\;\mathrm{of}\;\mathrm{four}\;\mathrm{measurements}\;\mathrm{all}\;\mathrm{valid}}{\mathrm{total}\;\mathrm{number}\;\mathrm{of}\;\mathrm{epochs}} \end{array}\right.$$

**Figure 8:**
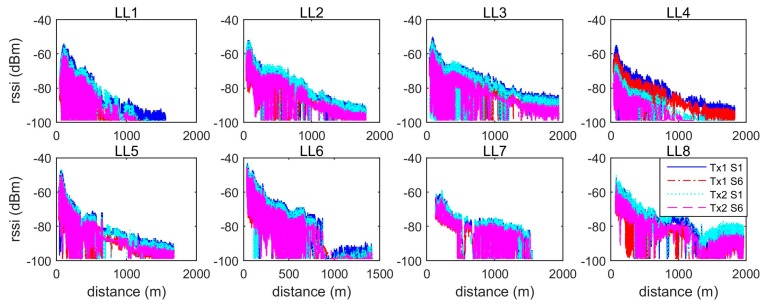
The Received Signal Strength Indicator (rssi) change (dBm) with respect to distance.

**Figure 9:**
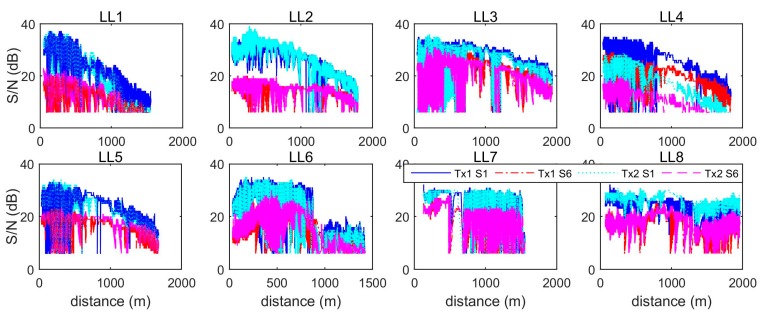
Trends of signal-to-noise ratio (dB) with respect to distance.

Figure 8 shows the Received Signal Strength Indicator (rssi) changes for each LL with respect to distance. Figure 9 shows the corresponding change trends for S/N (in dB). Based on a standard wireless signal propagation model, the rssi is a log function of the reciprocal of signal propagating distance. From Figure 8, it can be seen that the rssi values for LL1 to LL5 are consistent with this model, however not in the case of LL6 to LL8. Figure 9 shows similar change trends for the S/N, where the value of S/N for LL1 to LL5 linearly reduces as the distance increases. However, the change trend for LL6 to LL8 is more disordered due to significant signal interferences and blockages. Furthermore, the changes in S/N depend on the frequency, since the slopes for the S1 signal are steeper than for S6 signals, as shown in the panels for LL1, LL2, and LL5.

**Figure 10:**
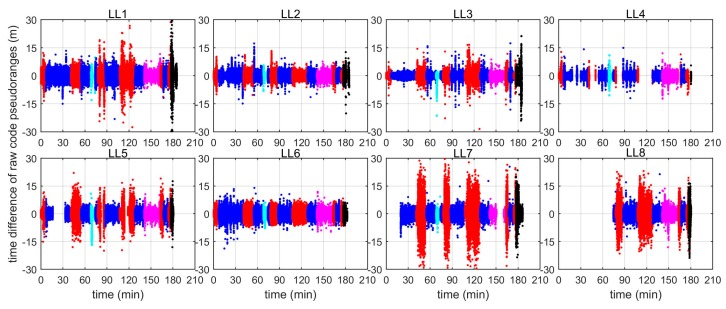
Time differences of raw code pseudorange measurements from each LL with geometry distance corrections (Unit: m).

To assess the stochastic characteristics of the code pseudorange measurements, the time differences of the raw code pseudorange measurements from each LL are plotted in Figure 10, with different colours indicating the measurements in different areas. The distance from the LocataLite transmitter to the rover has been subtracted, therefore only the measurement errors are left and are shown in Figure 10. As expected, the magnitudes of the measurement errors for each LL are different in different areas. In general, errors for measurements in area 1 (blue) and 4 (magenta) are much smaller than those in areas 2 (red), 3 (cyan), and 5 (black). Frequent and significant measurement outliers are observed in each LocataLite, and the largest measurement errors can reach 30 metres.

**Figure 11:**
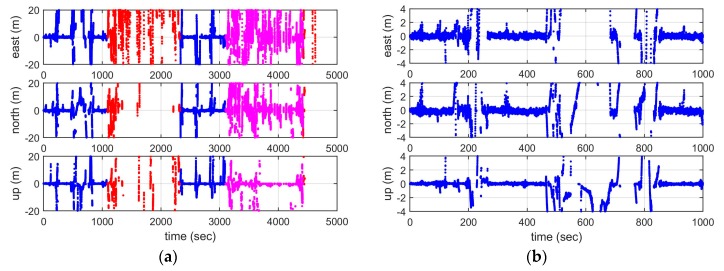
Positioning errors using raw code pseudorange measurements: (**a**) whole experiment, (**b**) the first 17 minutes.

Figure 11 shows the corresponding positioning errors of using the raw code pseudorange measurements, with the left panels indicating the positioning errors in the east, north, and vertical component during the whole test period, and the right panels showing the corresponding positioning errors during the first 17 minutes when the vessel travelled within area 1. Left panels show that the positioning accuracy is higher in area 1, and it is worst in area 2. The right panels show more detailed results for area 1, indicating that the general position errors are within 2 metres, both in a horizontal and vertical sense, except for some epochs with large measurement outliers.

**Figure 12:**
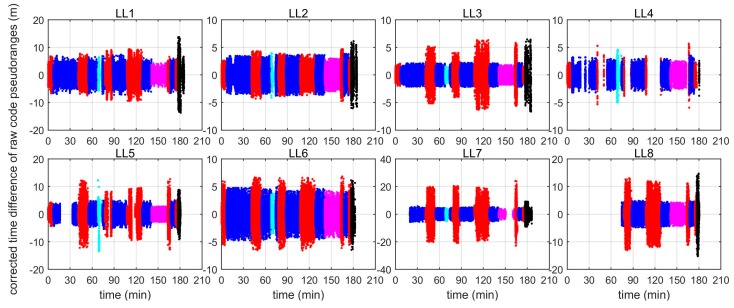
Time differences of code pseudorange measurements from each LL with geometry distance corrections after data preprocessing (Unit: m).

**Figure 13:**
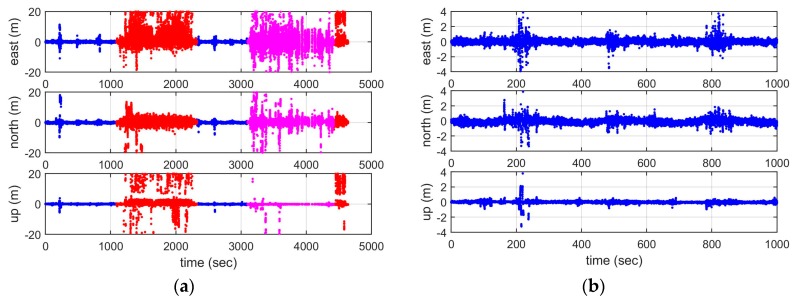
Positioning errors using code pseudorange measurements with robust estimation: (**a**) whole experiment, (**b**) the first 17 minutes.

The above analysis indicates that the Locata signal uncertainty is relatively high, so that precise positioning cannot be always guaranteed. Therefore, to improve the positioning accuracy, data preprocessing, as well as employing a robust positioning algorithm, is necessary. Similar to GNSS preprocessing, measurement outliers that contaminate raw measurements should be detected and excluded before being used for positioning. Figure 12 plots the time difference of the code pseudorange measurements after data preprocessing, which shows that the majority of measurement errors are within 5 m, and that errors in area 1 are obviously smaller than the errors in the other areas. Figure 13 shows the corresponding positioning errors of using data after preprocessing and then iteratively adjusting the measurement weights. When compared with the results in Figure 11, one can see that the positioning accuracy is significantly improved, especially in area 1, where the positioning errors are all less than 4 m, and 95% of them are less than 1 m.

### 5.3. Locata’s Challenging Issues

The implementation of Locata technology faces the following challenges, as can be concluded from the above discussion:

(1) Signal characteristics uncertainty. A precise mathematical model (including both functional and stochastic model) for Locata measurements is crucial in precise positioning. However, most existing studies focus on refining the functional model. Due to the spatiotemporal complexity of, and limited knowledge regarding, the signal characteristics, the current stochastic model is less well known.
(2) Installation complexity. To configure a LocataNet with spatial diversity, multiple antennas need to be mounted (Figure 2) on a concrete base, and the coordinates of the antennas have to be precisely surveyed [40]. There may be challenges regarding optimal configuration as well as power issues that are associated with a LocataNet installation.
(3) Environmental restrictions. The geometry of the LocataNet is restricted by the application environment. Furthermore, the VDOP is worse than the HDOP, and therefore constraining techniques are required to address the problem of vertical divergence of the positioning solutions.
(4) Extra hardware burden for the users. There is, as of yet, no ASIC design for the Locata Rover electronics, and hence the cost, bulk, and power demands are high.

## 6. Lessons Learned and Concluding Remarks

The Locata technology provides a solution to the demand for high-accuracy indoor and outdoor positioning, where GNSS cannot on its own provide the requisite capability. In this paper, some technical aspects of the technology were presented, including the critical technological components, the mathematical models for precise positioning, and the signal error sources. The literature describing a variety of applications of a Locata standalone system, a Locata/GNSS, and a Locata/GNSS/INS integration systems were reviewed, and significant challenges that may restrict its future application for custom-grade navigation in urban environments were discussed.

In summary, the major advantages of Locata technology are: (1) a flexible system and signal design that can prevent jamming and reduce the near-far problem under different signal environments; (2) the use of the TimeLoc technique ensures time synchronisation within a LocataNet as well as with respect to an external time scale (such as GNSS); (3) the installed LocataNet is easily expandable to increase the signal coverage; and, (4) the advanced VRay antenna design is very effective in mitigating multipath, and it provides angle measurements for more accurate position and attitude solutions. However, there are also some issues that may limit Locata’s future deployment, such as the relative high cost, incompatible user hardware, complex configuration of LocataNet, environmental constraints, and RFI. 

As far as academic research is concerned, future investigations should focus on classifying, testing, and modelling the various Locata signal errors and biases, as well as the integer ambiguity fixing algorithms, so as to improve the positioning accuracy and reliability of a Locata standalone system. Further research is also required for data fusion algorithms that more robustly integrate Locata measurements with those from other navigation sensors and systems, especially GNSS and INS.

## Figures and Tables

**Table 1:** Specification summary of Locata’s systems [16].

		First Generation System (Prototype Since 2002)	Second Generation System (Commercial Deployment Since 2006)
Signal structure	Frequencies	Single-frequency at GPS L1	Dual-frequency 2.4 GHz (80 MHz bandwidth)
	PRN code	C/A (1.023 MHz chipping rate)	Proprietary (10 MHz chipping rate)
	Licence requirements	Licensing issues & problem for wide area deployment	None required, FCC compliant
LocataLite (transceiver)	Hardware	FPGA & DDS technology	FPGA & DDS technology with a modular design
	Output power	Several microwatts	Maximum of 1 watt
	Range	~600 metres	~10 km line-of-sight
	Antenna	RHCP patch & ¼ wave	Antenna design dependent on application
	Size	260 × 200 × 45 mm	240 × 135 × 30 mm
	Weight	2.1 kg	1 kg
Locata Rover (receiver)	Hardware	Zarlink/Mitel based GPS receiver chipset	FPGA technology, modular design
	Measurement rate	1 Hz	25 Hz
	RT positioning	1 Hz on-board	25 Hz through LINE software, 10 Hz onboard
	AR	Known point initialisation (KPI)	On-the-fly (OTF)
	Antenna	Various types tested including RHCP patch and ¼ wave	Antenna design will depend on application
	Size	200 × 100 × 40 mm	130 × 135 × 30 mm
	Weight	300 g	500 g
